# Molecular mechanisms of surface antigen suppression by ApiAP2 and its implications for vaccine development

**DOI:** 10.1186/s13567-025-01491-2

**Published:** 2025-03-22

**Authors:** Peiyao Li, Rina Su, Ganglin Ren, Hongbin Si, Xingju Song, Dandan Hu

**Affiliations:** 1 Guangxi Key Laboratory of Animal Breeding, Disease Control and Prevention, College of Animal Science and Technology, Guangxi University, Nanning, 530004 China; 2 Guangxi Zhuang Autonomous Region Engineering Research Center of Veterinary Biologics, Nanning, 530004 China

**Keywords:** ApiAP2, surface antigen, *Eimeria*, vaccine

## Abstract

The apicomplexan parasite *Eimeria* is the causative agent of coccidiosis, which results in enteritis in animals and imposes a significant economic burden on the farming industry. Apicomplexan Apetala2/ERF (ApiAP2) transcription factors are known to play crucial roles in various processes of Apicomplexa parasites. However, little is understood regarding the role of ApiAP2s in *Eimeria* species. In this study, we performed a functional characterisation of ApiAP2 through CRISPR-Cas9-mediated knockout. Our results revealed that the parasite’s invasion efficiency, total oocyst output, and virulence were significantly impaired after EtAP2-S1 depletion. RNA sequencing (RNA-Seq) and Cleavage Under Targets and Tagmentation (CUT&Tag) analyses showed that EtAP2-S1 targets the promoters of numerous genes, and its knockout resulted in the up-regulation of 59 *sag* genes. Furthermore, the knockout strain exhibited significantly lower virulence while offering excellent immune protection, making it a promising candidate for a vaccine. This study demonstrates that EtAP2-S1 is a fitness-conferring gene that suppresses the expression of *sag* genes in *E. tenella*, which is the first step in developing gene-knockout vaccines to control coccidiosis.

**Supplementary Information:**

The online version contains supplementary material available at 10.1186/s13567-025-01491-2.

## Introduction

Coccidiosis, caused by the apicomplexan parasite *Eimeria* species, results in haematochezia, reduced feed efficiency, and mortality in farm animals, especially chickens [1]. Chicken coccidiosis is prevalent worldwide and causes substantial economic losses to the poultry industry. It is estimated to cost the poultry industry more than £10 billion annually [2].

The life cycle of the *Eimeria* species encompasses three critical stages: sexual gametogony, asexual schizogony, and sporogony [3]. Endogenous development comprises both asexual and sexual reproduction within host cells, whereas exogenous sporogony development occurs externally. The *Eimeria* life cycle begins when the host ingests infectious sporulated oocysts. These oocysts release sporozoites into the digestive tract, where they invade the intestinal epithelial cells. Through asexual reproduction, the invading sporozoites produce numerous merozoites that go on to infect other host cells. After three to five rounds of asexual amplification, all parasites undergo sexual development, producing micro- and macro-gametocytes and eventually completing fertilisation. The fertilised zygotes develop into unsporulated oocysts, which are then excreted with faeces, signalling the beginning of the exogenous sporogony phase. This process makes the oocysts infectious with the sporozoites inside [4, 5].

Maintaining parasitism and transitioning between stages depend on the precise transcriptional and translational regulation of the parasite genes. This process potentially involves key regulatory factors, which offer potential targets for intervention strategies [6–8]. The plant-like apicomplexan Apetala AP2 (ApiAP2) transcription factors have been shown to be crucial in regulating many biological processes, including virulence, cell cycle transition, and stage conversion [9, 10]. Multiple ApiAP2s are involved in the tachyzoite-bradyzoite transition in *Toxoplasma gondii*, including TgAP2XI-4 [11], TgAP2IX-4 [12], TgAP2IV-3 [13], TgAP2IV-4 [14], and TgAP2IX-9 [15]. 

Recently, TgBFD1, another Myb-like transcription factor, has been identified as a master regulator of chronic-stage differentiation [16]. Since TgBFD2 is a direct target of TgBFD1, translating the TgBFD1 gene is also necessary due to the interaction with a cytosolic RNA-binding protein TgBFD2 in a feedback loop [17]. Gametocytogenesis is essential for malaria transmission, and the transcription factor AP2-G and other epigenetic modulators have been shown to control sexual commitment to this process [18–20]. In *Toxoplasma*, cat (the only definitive host) stage-restricted genes are suppressed by the MORC-HDAC3-AP2s remodelling complex during the intermediate host’s tachyzoite stage [21–25]. TgAP2XII-1 and TgAP2XI-2 form heterodimers that bind to the promoters of merozoite-specific genes and recruit MORC and HDAC3. Disruption of this suppression protein complex results in stage conversion and the production of merozoites in vitro [22, 23].

A recent transcriptomic study by Chen et al. [8] identified 53 ApiAP2s expressed in different stages of *E. tenella*. These ApiAP2s can be classified into four clusters: those expressed mainly in the unsporulated and sporulating oocyst stage, the merozoite stage, the sporulated oocyst stage, and the gametocyte stage [8]. The study also characterised a sporogony-specific ApiAP2 ETH2_0411800 and found that the overexpression of this gene did not influence parasite development. In contrast, a significant decrease in oocyst output was observed when it was completely knocked out [8]. In our previous study, we performed a preliminary loss-of-function screening for 33 ApiAP2s in *E. tenella* and demonstrated that 10 of them were dispensable [26]. However, the functions and roles of most *E. tenella* ApiAP2s in parasite development remain unknown.

Here, we characterised the *E. tenella* ortholog of TgAP2XI-2 and found that it suppresses the expression of multiple surface antigens (SAGs) and is important for parasite growth and virulence. Additionally, the knockout strain exhibited good performance as a vaccine candidate with low pathogenicity and high immunogenicity. This study offers crucial insights into understanding coccidia parasites’ gene regulation and molecular biology and provides the first gene-knockout vaccine candidate for coccidiosis.

## Materials and methods

### Ethics statement

All animal experiments in this study were approved by the Administration Committee of Laboratory Animals at Guangxi University and were conducted following the guidelines of the Institutional Animal Care and Use Committee (Approval number: GXU-2022-265).

### Animals and parasites

The *E. tenella* Houghton strain, which stably expresses Cas9 (EtHCYA) [26], was used to construct knockout strains. The wild-type *E. tenella* Houghton (EtH) strain was used for gene overexpression strain construction. These strains were maintained and propagated through one- to four-week-old sanhuang chickens. Chickens were reared in a coccidia-free environment and provided with water and feed free of coccidiostats and antibiotics. Parasites were collected, purified, and sporulated as described previously [27].

### Plasmid construction

The gene knockout (KO) plasmid containing the U6-gRNA cassette and the dihydrofolate reductase (DHFR)-mCherry positive selectable cassette was constructed as previously reported [26]. Briefly, the small guide RNA was designed using the Eukaryotic Pathogen gRNA Design Tool (EuPaGDT) and the gRNA in the Yellow Fluorescent Protein (YFP)-KO template vector was replaced by a seamless assembly strategy (ClonExpress MultiS One Step Cloning Kit, Vazyme biotech co., Ltd, Nanjing). In the knockout plasmid, the mCherry sequence was fused to the pyrimethamine-resistant gene, *TgDHFR-ts-m2m3* (DHFR), and was expressed under the control of the EtMic2 promoter. In contrast, the gRNA was derived from the *E. tenella* U6 promoter. The coding sequence of ETH2_0734800 (referred to as EtAP2-S1 hereafter) was amplified from the cDNA of *E. tenella* sporozoites and inserted into the Flag-tagged overexpression vector to overexpress EtAP2-S1. The expression of EtAP2-S1-Flag was regulated by the 5′- and 3′-UTR of EtActin. The promoter of EtMic2 derived the other DHFR-EYFP expression cassette. All polymerase chain reaction (PCR) amplifications were performed using Phanta Max Master Mix (Vazyme Biotech Co., Ltd, Nanjing). All primers used in this study are listed in Additional file 1.

### Transfection and establishment of the knockout and overexpression lines

The transgenic procedures for *Eimeria* species were performed with modifications based on our previous report [28]. Briefly, sporocysts of EtHCYA or EtH were prepared using freshly purified oocysts by a 50% Percoll (Solarbio, Beijing, China) density gradient following glass bead grinding. The sporocysts were then excysted in bile-trypsin excystation buffer at 42 °C for 60 min. The knockout or overexpression plasmids were linearised by incubation with SnaBI at 37 °C for 60 min. Subsequently, ~1 × 10^7^ sporozoites were transfected with 20 μg of each linearised plasmid in a cytomix buffer in a final volume of 100 μL. Transfection was executed using the Nucleofector 2B (Lonza) and the U-033 program, with transfected parasites inoculated via the cloaca in five two-week-old chickens. Positive transfectants were selected using multiple rounds of selection, with pyrimethamine (150 ppm, Aladdin, Shanghai, China) treatment and flow cytometry (FACSAria II, BD, USA). The single oocyst of the EtAP2-S1 knockout strain was separated and then isolated to generate stable lines.

### Indirect immunofluorescence assay

IFA experiments were conducted to identify the protein localisation of EtAP2-S1 using the overexpression line. Sporozoites were prepared as described above. Fresh extracellular sporozoites were mounted onto polylysine-treated cell slides, fixed with 4% formaldehyde for 10 min, and then permeabilised with 0.25% Triton X-100 in phosphate-buffered saline (PBS) for 8 min. Subsequently, the parasites were stained with mouse anti-Flag antibody (1:500, Abmart, Shanghai, China) and rabbit anti-TgIMC1 polyclone antibody (against the ortholog genes in *E. tenella*, and staining for the parasites’ inner membrane complex) for 1 h at 37 ℃ after being blocked with 3% bovine serum albumin (BSA) for 30 min. After several washes with PBS, the slides were coated with Cy3-conjugated goat anti-mouse IgG (1:100, Huaxinbio, Beijing, China) for 1 h at 37 ℃. The nuclei were stained with Hoechst 33,258 (1:100, Sigma, St. Louis, MO, USA). Images were obtained using a Zeiss Fluorescence Microscopy system (Zeiss, Germany).

### Immunoblotting

Immunoblotting confirmed the expression of EtAP2-S1 in its overexpression line. Total sporozoite proteins from the EtAP2-S1 overexpression and wild strains were extracted by lysis with RIPA buffer (Huaxinbio, Beijing, China) on ice for 30 min. Protein samples were then separated in 12% SDS-PAGE and transferred to nitrocellulose membranes (Millipore, Bedford, MA, USA). Subsequently, the membranes were blocked with 5% skimmed milk for 1 h at 37 ℃, followed by overnight incubation with mouse anti-Flag mAb (1:1000, Abmart, Shanghai, China) or Anti-Actin mouse mAb (1:2000, Proteintech, USA) at 4 °C. After washing thrice with phosphate-buffered saline with Tween (PBST), the membranes were incubated with horseradish peroxidase (HRP)-conjugated goat anti-mouse IgG (1:1000 dilution, Huaxinbio, Beijing, China) for 1 h at 37 °C. The proteins were then visualised using chemiluminescent reagents (Huaxinbio, Beijing, China) and ChemiDoc XRS + (Bio-Rad, Hercules, CA, USA).

### Oocyst output curve and total oocyst output

Five seven-day-old Sanhuang broilers were infected with 1000 fresh oocysts/bird for each strain. Using an advanced McMaster counter, the oocyst output was counted daily for 5–12 days post-infection (dpi). All faeces were collected at 5–12 dpi for total oocyst output counting. All detections were performed at least three times.

### Virulence

Five Sanhuang chickens at seven days old were infected with 2 × 10^4^ or 5 × 10^4^ fresh sporulated oocysts/bird for each strain. For the average body weight gain measurements, chickens were weighed at day 0 and 10 dpi. For intestinal lesion scoring, chickens were sacrificed at 7 dpi, with the ceca removed for lesion scoring, following the rules described by Johnson and Reid [29]. Furthermore, total oocyst output was also counted for these experiments.

### Endogenous development analysis

Chickens were infected with 1 × 10^4^ sporulated oocysts/bird of the EtHCYA or EtAP2-S1 knockout *E. tenella* strains. The ceca of the chickens were removed during 4–7 dpi at 0.5-day intervals. The ceca sections were stained with haematoxylin and eosin staining (H&E) and observed under a microscope. The average number of schizonts in 1 cm^2^ of the intestine and the average size of schizonts were measured by counting at least 20 random images/schizonts.

### Invasion assay

Freshly harvested sporozoites (5 × 10^5^) of EtHCYA or EtAP2-S1 knockout strains were used to infect well-developed DF-1 cells in a 12-well plate for 2, 4, 8, and 12 h, respectively. Uninvaded parasites were then removed by washing thrice with PBS, and all uninvaded sporozoites in each well were counted using a haemocytometer. The invasion rates = (total number of inoculated sporozoites−uninvaded sporozoites)/total number of inoculated sporozoites × 100%. These experiments were repeated in triplicates.

### Sporulating assay

Unsporulated oocysts from each strain were purified from the cecal contents by flotation in a saturated salt solution after 7 dpi. Unsporulated oocysts were then counted and diluted to 5 × 10^5^/mL in 2.5% K₂Cr₂O₇ solution before being incubated in a shaking incubator at 28 °C and 160 rpm. The sporulated and unsporulated oocysts were counted at 12-h intervals, and the sporulation rates were calculated. Counts were performed in triplicates.

### Immuno-protection experiments

Forty seven-day-old Sanhuang chickens were randomly selected and divided into four groups: the unimmunised and unchallenged group (UUC), the unimmunised and challenged group (UCC), the EtHCYA immunised group, and the EtAP2-S1-KO immunised group. The birds were orally exposed to 1000 fresh sporulated oocysts from different strains and challenged at 14 dpi with 2 × 10^4^ sporulated oocysts from the EtHCYA parent line. The intestinal lesions were examined five days post-challenge, with the total oocyst output and average body weight gains measured as described above. Finally, the anti-coccidia index (ACI) was calculated as previously reported [30].

### RNA-Seq and data analysis

Total RNAs from the sporozoites of EtHCYA and EtAP2-S1-KO strains were isolated using Trizol regent. The genomic DNA was removed by DNase I (Tiangen Biotech Co., Ltd, Beijing, China). The purity, concentration, and integrity of RNAs were tested using the NanoPhotometer^®^ (Implen, CA, USA), the Qubit^®^ RNA Assay Kit in Qubit^®^ 2.0 Fluorometer (Life Technologies, CA, USA), and the RNA Nano 6000 Assay Kit of the Bioanalyzer 2100 system (Agilent Technologies, CA, USA), respectively. Sequencing libraries were generated using the Truseq™ RNA Sample Prep Kit (Illumina, CA, USA) according to the manufacturer’s instructions. Sequencing was then performed using the Illumina NovaSeq 6000 platform to generate 150 bp paired-end reads (Novogene, Beijing, China).

Pair-end clean reads were aligned to the *E. tenella* reference genome [31] (ToxoDB.org, release 68) using Hisat2 version 2.2.1 [32]. The output SAM files were converted into BAM files, sorted and indexed using Samtools [33]. The BAM files were then used for read count via htseq-count version 0.13.5 [34]. The differentially expressed genes (DEGs) between the groups were calculated by the R package DEseq2 [35]. Functional enrichment analysis (Gene Ontology (GO) and Kyoto Encyclopaedia of Genes and Genomes (KEGG)) was performed using ClusterProfiler (v 4.0.5) [36]. Gene expression with a |Log_2_Fold change|≥ 2 and an adjusted *P* value < 0.01 was deemed significantly differentially expressed. Transcripts per million (TPM) were calculated for each gene and used for clustered heatmap drawing.

### Cleavage Under Targets and Tagmentation and data analysis

Fresh sporozoites of EtH and EtAP2-S1-OE strains (1 × 10^7^ for each experiment) were prepared as described above. Per the manufacturer’s instructions, library construction was conducted using formaldehyde cross-linking Cleavage Under Targets and Tagmentation (CUT&Tag) with the NovoNGS® CUT&Tag 4.0 High-Sensitivity Kit (for Illumina®) (Novoprotein, Nanjing, China). Briefly, fresh sporozoites were treated with 1% formaldehyde for 10 min at room temperature. The cross-link was terminated by 1 × Glycine solution. The sporozoites were washed, bound to activated concanavalin A beads (10 μL/sample), and incubated for 10 min at room temperature. The mixture was then resuspended and incubated overnight with a primary antibody (1:50, mouse anti-Flag mAb, Abmart, Shanghai, China) at 4 °C. After several washes, the parasites were incubated with a secondary antibody (1:100, goat anti-mouse IgG, Proteintech, USA) for 1 h at room temperature. Parasites were then resuspended with 65 μL of Tagmentation Buffer (ChiTag pAG-Tn5 buffer + 1 μL of 0.7 M MgCl_2_) and incubated for 1 h at room temperature on a rotator. Tagmentation was stopped by 5 μL of stop buffer and 1 μL of proteinase K at 55 °C for 2 h. The DNA extraction was performed using Tagment DNA Extract Beads. The Illumina sequencing libraries were generated through PCR amplification using specific adaptors per the manufacturer’s recommendations. The CUT&Tag libraries were sequenced using the Illumina NovaSeq 6000 platform (Novogene, Beijing, China).

The paired-end reads were filtered and aligned to the *E. tenella* reference genome (ToxoDB.org, release 68) using Bowtie2 (v.2.1.0) [37]. The sorted BAM files were cleaned of unmapped or low-quality reads and PCR duplicates using Samtools [33] and Picard, respectively. Filtered reads were then employed to identify CUT&Tag peaks using MACS2 [38]. The overlapping peaks in the two biological replicates were determined by the Irreproducible Discovery Rate (IDR) [39]. Subsequently, the final peaks were annotated against the *E. tenella* reference genome. The sorted and filtered BAM files of CUT&Tag peaks and RNA-Seq reads were normalised to reads per kilobase million (RPKM) with a resolution of 10 bp (bin size) and transformed into bigwig files for direct visualisation in the Integrative Genomics Viewer (IGV) [40].

### Quantitative polymerase chain reaction

Quantitative PCR (qPCR) was performed to identify the transcription pattern of EtAP2-S1. Subsequently, the cDNA samples were synthesised from DNase-treated RNAs extracted from unsporulated oocysts, sporulated oocysts, and merozoites. PCR reactions were conducted on the Roche LightCycler^®^ 480 system using the TransScript^®^ II Green One-Step qRT-PCR SuperMix (TransGen Biotech, Beijing). Reactions were carried out in triplicate for each sample. The primers used are listed in Additional file 1. As reported previously, the expression of each gene was normalised to the reference gene glyceraldehyde 3-phosphate dehydrogenase (GAPDH) [41].

### Statistical analysis

Unpaired multiple *t*-tests were employed to analyse total oocyst output, weight gain, lesion score, and sporulation rate. Two-way analysis of variance (ANOVA) was used in the invasion rate, oocyst output curve, and virulence studies. All bar plots depict the mean, with standard deviations indicated by error bars.

## Results

### Sequence features and expression pattern of EtAP2-S1

Previous studies have identified that TgAP2XII-1 and TgAP2XI-2 play vital roles in regulating the gene expression of *T. gondii* merozoite, along with the stage conversion [22–25]. Therefore, to ascertain whether orthologous genes exist in its relative species *E. tenella* and to understand their roles in the coccidia life cycle, we conducted a Basic Local Alignment Search Tool (BLAST) search using the complete amino acid sequences of TgAP2XII-1 and TgAP2XI-2 against other coccidia genomes. Our findings indicated that ETH2_0940300 showed the highest identity to TgAP2XII-1 (E-value = 1e−51), while ETH2_0734800 exhibited the highest identity to TgAP2XI-2 (E-value = 1e−20). In our previous study, we tried to knockout ETH2_0940300 but were unsuccessful, suggesting that it may be essential for the coccidia life cycle [8]. Thus, in this study, we focused on ETH2_0734800 (named EtAP2-S1).

**Figure 1:**
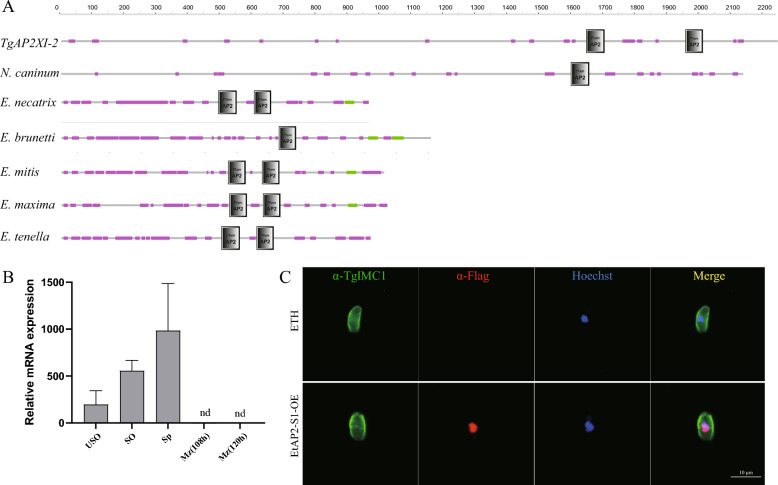
**Structure and expression pattern of EtAP2-S1.** **A** Diagrams showing the domain structures of ApiAP2s. **B** Quantitative PCR analysis of the relative transcription of EtAP2-S1 in different stages of *E. tenella*. USO, unsporulated oocyst; SO, sporulated oocyst; SP, sporozoite; MZ, merozoite (108 hpi). Nd, not detectable. **C** Protein localisation of EtAP2-S1. Immunofluorescent assay (IFA) was performed using an anti-Flag antibody with EtAP2-S1 overexpression parasite. The parasite membrane was stained with anti-TgIMC1, and the nucleus was stained with Hoechst.

Furthermore, through sequence analysis, we found that two AP2 domains (502-558 aa, 612-664 aa) were encoded by EtAP2-S1 and presented a high identity to the ortholog genes in other *Eimeria* species (Figure 1A). Here, we used qPCR to analyse the transcription level of EtAP2-S1 during the *Eimeria* life cycle. The results indicated that EtAP2-S1 was highly expressed in the sporulated oocysts and sporozoites compared to the unsporulated oocysts, but it was not detectable in the endogenous schizogony stages (Figure 1B).

To corroborate the protein localisation of EtAP2-S1, we constructed an overexpression strain of EtAP2-S1 (EtAP2-S1-OE) by randomly inserting an additional Flag-tagged copy (Additional files 2A, B). This strain was validated using PCR, and the expression of Flag-tagged EtAP2-S1 was confirmed by western blotting (Additional files 2C, D). The oocyst output and total oocyst output per bird of EtAP2-S1-OE were not significantly different from those of the wild type (Additional files 2E, F). Immunofluorescence staining using the anti-Flag antibody showed that the EtAP2-S1 co-stained with the nucleus marker Hoechst and localised to the nucleus (Figure 1C), which is consistent with its function as a transcription factor.

### EtAP2-S1 knockout impairs parasite endogenous development and invasion

**Figure 2:**
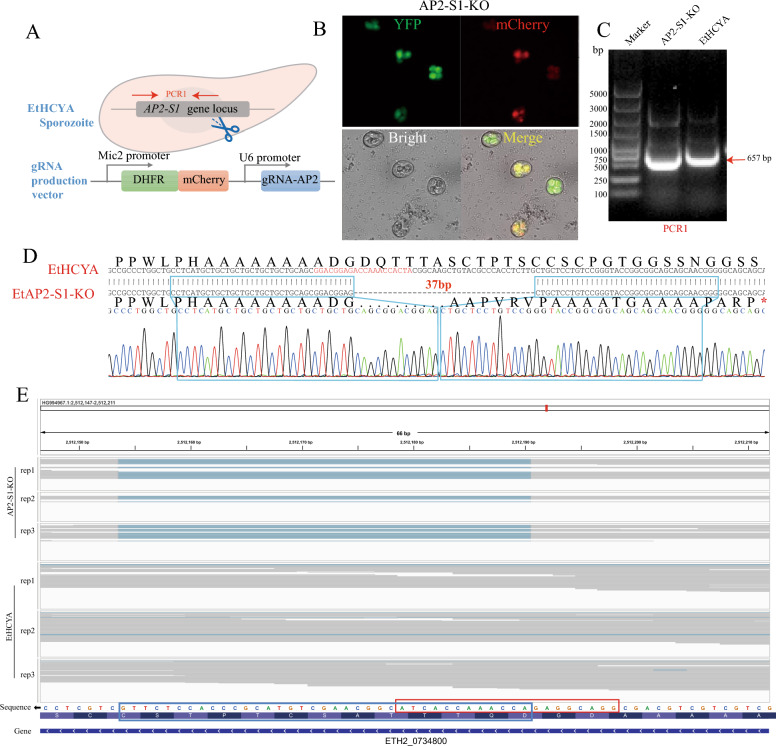
**Construction and identification of EtAP2-S1 knockout strain.** **A** Schematics showing the knockout strategy and plasmid elements. Cas9-expressing sporozoites were transfected using a linearised gRNA production vector targeting the N-terminal end of the EtAP2-S1 coding region. **B** Fluorescence microscopy image of EtAP2-S1-KO stain. **C** PCR identification of EtAP2-S1-KO stain. **D** Sanger sequencing and chromatogram showing the 37 bp deletion in EtAP2-S1-KO stain. The gRNA in EtHCYA DNA is marked in red. The stop codon was marked as a red asterisk. **E** IGV screenshots of Illumina sequencing reads showing the 37 bp deletion (blue frame) in different repeats of EtAP2-S1-KO stain. RNA-Seq was performed using sporozoites of EtAP2-S1-KO and EtHCYA stains (3 biological repeats each) with gRNA marked in a red frame.

We endeavoured to directly delete this gene to better understand the function of EtAP2-S1 in parasite biology by transfecting the Cas9-expressing line with a gRNA-producing plasmid, as demonstrated in our previous study (Figure 2A) [26]. Through several generations of pyrimethamine-and-fluorescent selection, followed by a final step of single oocyst isolation, we obtained a single clone of the EtAP2-S1 knockout *E. tenella* strain (EtAP2-S1-KO, Figure 2B).

In this study, we found that the deletion in the genome of the EtAP2-S1-KO strain occurs in the protein-coding region (185–221 bp, 37 bp in length). This deletion results in the stop codon gaining and the early termination of translation, ultimately depleting the EtAP2-S1 protein. This outcome was validated by PCR and Sanger sequencing (Figures 2C and D) and further substantiated by reads coverage in Illumina RNA-Seq (Figure 2E). However, this result is inconsistent with our previous loss-of-function screening, which may have lacked sufficient power because only one repeat transfection was performed [26].

**Figure 3:**
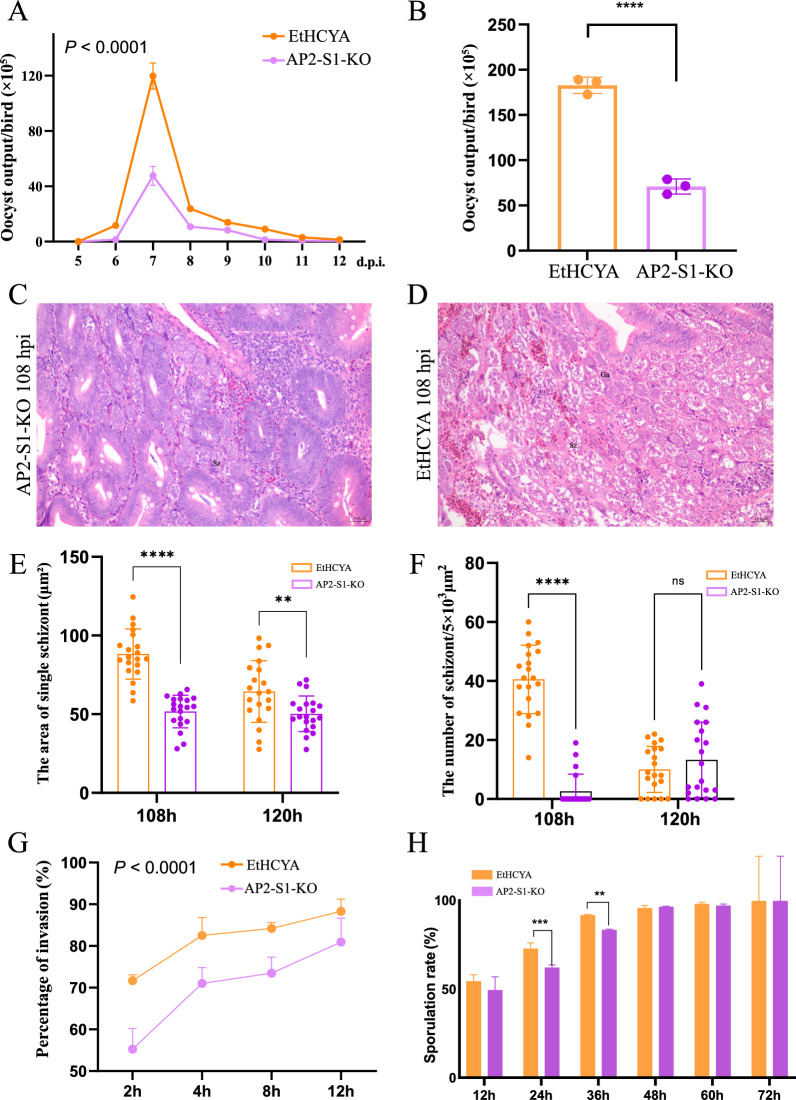
**Biological characterisation of EtAP2-S1-KO stain.** **A** Oocyst output curves of EtAP2-S1-KO and EtHCYA strains. Chickens (*n* = 5) were infected with 1000 fresh oocysts/bird for each strain, and oocyst outputs were monitored daily during 5-12 dpi. Total oocyst output was calculated for each bird (**B**). Representative images of H&E stained cecal sections of 108 hpi chickens with EtAP2-S1-KO (**C**) or EtHCYA (**D**) strains. The average size of the schizonts (**E**) and the average number of schizonts per cm^2^ (**F**) were monitored based on 20 random images from the HE sections of 108 hpi and 120 hpi. **G** Comparative invasion efficacy of the sporozoites of EtAP2-S1-KO and EtHCYA strains. Sporozoites were inoculated into DF-1 cells at different times, and the uninvaded parasites were washed out for counting. **H** Sporulation efficacy. Freshly purified unsporulated oocysts were incubated at 28 °C and 160 rpm, and then the sporulation rate was calculated at 12 h intervals. ***P < 0.001; ****P < 0.0001.

The endogenous development of coccidia involves several processes, such as invasion, intracellular replication, and sexual fertilisation [5]. Here, the oocyst output curve of the gene-knockout strain was measured to characterise these processes. We found that the EtAP2-S1-KO strain had a prepatent time of 136 h post-infection (hpi), and the oocyst output peaked at 7 dpi. These findings were identical to the parent strain (Figure 3A). However, a significant decrease in the daily oocyst output was observed, especially for the oocyst output at their peaks (Figure 3A). Consequently, the total oocyst output of the knockout strain was reduced by ~61% (Figure 3B). Additionally, endogenous schizogony development was observed through HE staining of the intestinal sections after being infected with EtAP2-S1 knockout or EtHCYA stains at different time points (Figures 3C and D).

By counting the size and number of schizonts in the sections of the EtAP2-S1-KO strain after 108 and 120 hpi (the time points abundant for the second and third generation of schizonts, respectively), we found that they decreased in size and number after 108 hpi (Figures 3E and F). In contrast, no significant difference was found after 120 hpi due to the reduced total number of schizonts and increased gametes. These results suggest a significant growth defect for endogenous development after EtAP2-S1 depletion.

The invasive ability of sporozoites was measured in vitro using a chicken embryo fibroblast cell (DF-1) model. Fresh sporozoites were allowed to infect DF-1 cells at different times, after which the uninvaded parasites were washed and collected for counting. The results showed that the invasion rates rose with increasing exposure time for both strains. However, the knockout strain consistently had a significantly lower invasion rate than its parent strain (Figure 3G). After only 2 h of exposure, more than 70% of EtHCYA sporozoites invaded the host cells, whereas, for the EtAP2-S1-KO strain, only 55% of sporozoites invaded (Figure 3G). These results suggest that EtAP2-S1 knockout significantly decreases the invasive ability of the parasite.

While we also observed the exogenous sporogony of the parasites, no significant differences were recorded in the final sporulation rates except at certain time points (24 h and 36 h) (Figure 3H).

### EtAP2-S1 knockout significantly reduces parasite virulence

After demonstrating that endogenous development and invasive ability were impaired following EtAP2-S1 depletion, we speculated that the virulence of the EtAP2-S1-KO strain could also be influenced. Thus, we compared the virulence of the knockout strain and its parent strain. Consequently, seven-day-old chickens were infected with different doses of fresh sporulated oocysts from EtAP2-S1-KO or EtHCYA. Cecal lesions were observed after 7 dpi, and significant differences were found between EtAP2-S1-KO and EtHCYA strains.

**Figure 4:**
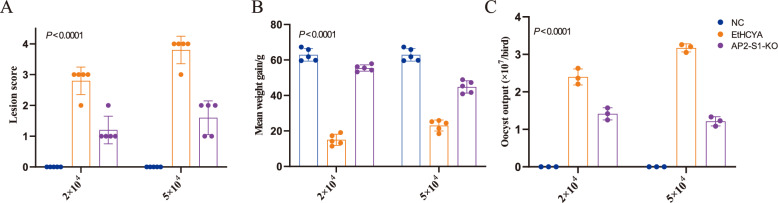
**EtAP2-S1 knockout reduces parasite virulence.** Each chicken was infected with 2 × 10^4^ or 5 × 10^4^ oocyst from the EtAP2-S1-KO or EtHCYA strain. The average body weight gain (**A**), intestinal lesion scores (**B**), and total oocyst outputs (**C**) were measured.

The findings indicated that even in the high infection dosage group, the chickens infected with EtAP2-S1-KO had lesion scores ranging from 1 to 2 points (see Figure 4A). Nonetheless, most chickens in the low infection dosage group were assigned a score of 3 points, while those in the high infection dosage group received 4 points (see Figure 4A). Furthermore, the average body weight gains were measured after 10 dpi. Our findings indicated that EtHCYA infection led to a dramatic decrease in body weight compared to uninfected controls (Figure 4B). Statistically significant yet minor differences were observed between the EtAP2-S1-KO-infected group and the uninfected controls, as well as substantial differences between the infected groups (Figure 4B). Additionally, the total oocyst output was significantly lower in the EtAP2-S1-KO strain than in EtHCYA (Figure 4C), which is consistent with our abovementioned results. These results demonstrate a significant reduction in parasite virulence after EtAP2-S1 knockout.

### EtAP2-S1 knockout increases surface antigen expression in sporozoites

**Figure 5:**
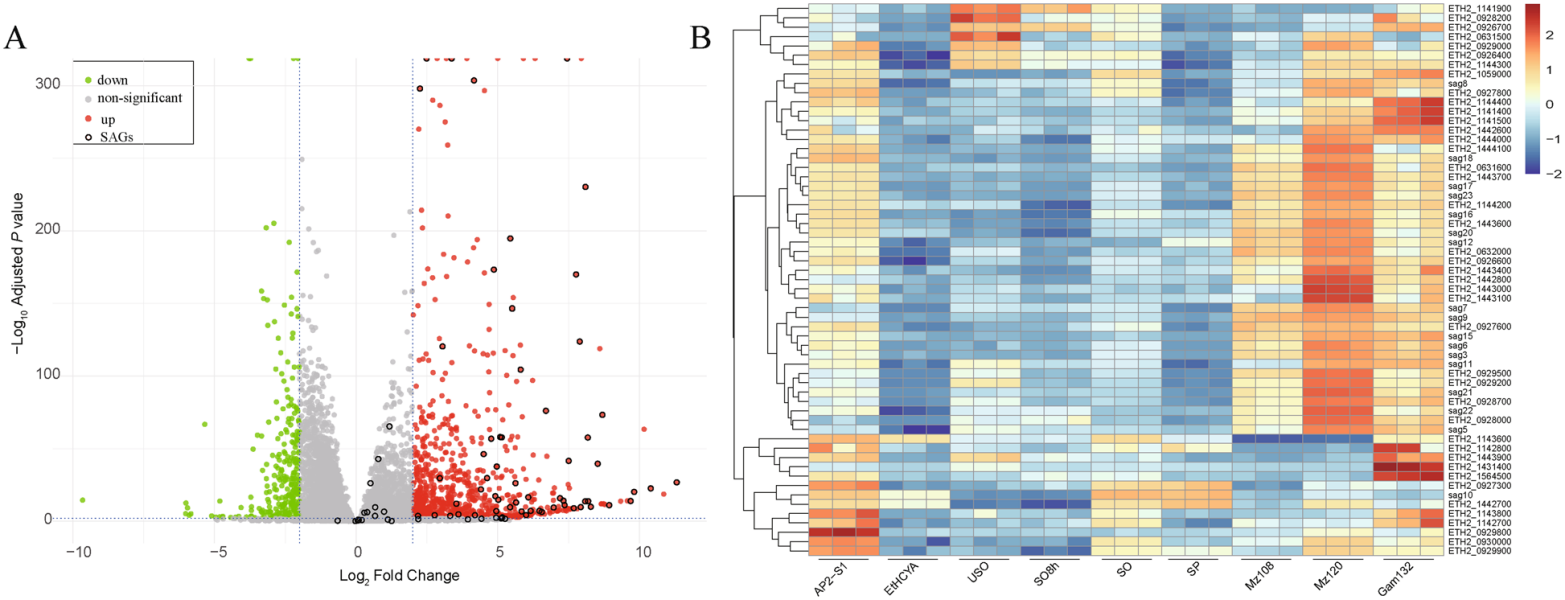
**Transcriptome analysis of EtAP2-S1-KO strain.** **A** Volcano plot of differentially expressed genes (*P* < 0.01, |log_2_FC|≥ 2). Green and red plots show downregulated and up-regulated genes, respectively. Black circled dots represent *sag* gene families. **B** Heatmap for the differentially expressed *sag* gene families. The parasite lifecycle data were generated by Chen et al. [8]. USO, unsporulated oocyst; SO8h, sporulating oocyst; SO, sporulated oocyst; SP, sporozoite; MZ108, merozoite (108 hpi); MZ120, merozoite (120 hpi); Gam132, gametocyte (132 hpi).

Considering the gene transcription regulation role of ApiAP2s, we performed an RNA-Seq of EtAP2-S1-KO and EtHCYA sporozoites. Through DESeq2 analysis with |log_2_fold change|≥ 2 and an adjusted *P* value < 0.01, 1097 DEGs were identified, comprising 816 up-regulated and 281 down-regulated (Figure 5A; Additional file 3). Although the *sag* gene family is generally acknowledged as *Eimeria’s* major surface antigen gene family, its function is not fully understood. Evidence suggests it may be involved in parasite attachment, invasion, and host-parasite interactions [42]. Moreover, in *E. tenella*, 89 surface antigen genes (SAGs) have been identified according to its N-terminal signal peptide and membrane-bound feature tethered by glycosylphosphatidylinositol (GPI) anchors at the C-terminal [43]. Here, we found that 59 (66.3%) of them were up-regulated after EtAP2-S1 knockout (Figures 5A and B; Additional file 3), which reveals a significant effect of EtAP2-S1 on the transcription of *sag* gene families.

In addition, the differential expression of many zinc-containing proteins was noted, of which 13 were down-regulated, and 11 were up-regulated (Additional file 4A). Furthermore, 20 genes associated with gametocyte development were up-regulated, including the motor protein dynein, cilia- and flagella-associated proteins for the microgametes, as well as the subtilisin-like protease for the macrogametes (Additional file 4B). However, the majority of these differently expressed gamete and zinc figure genes maintain very low expression levels, making their role in the development of the EtAP2-S1-KO parasite ambiguous.

### EtAP2-S1 binds to the upstream region of *E. tenella* protein-coding genes

**Figure 6:**
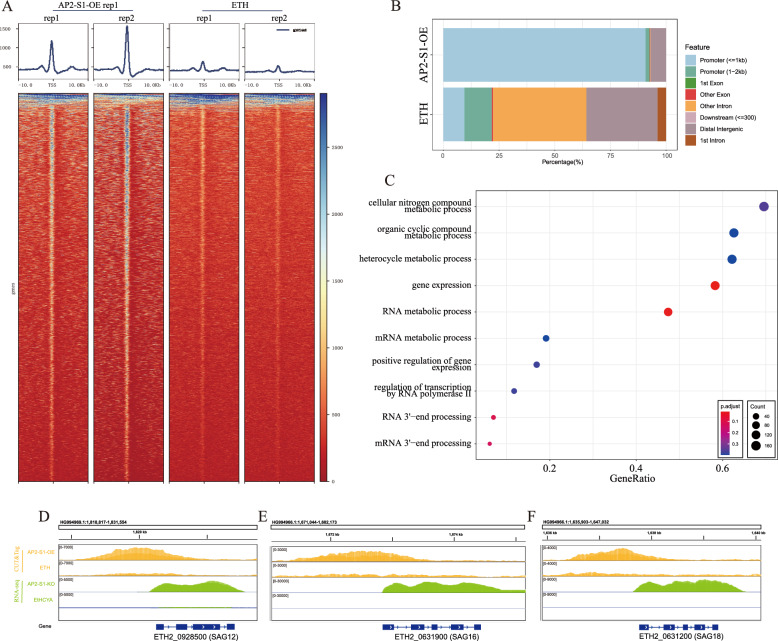
**EtAP2-S1 occupancy on E. tenella genome revealed by CUT&Tag enrichment.** Sporozoites of EtAP2-S1 overexpressing strain and wild-type strain were used for enrichment using anti-Flag antibody. **A** Heatmap and profile analysis of the CUT&Tag peak intensity for EtAP2-S1. TSS, transcription start site. **B** Global distribution of significant peaks within genomic features. **C** Gene ontology enrichment of EtAP2-S1 targeted genes. **D**–**F** Representative IGV screenshots of overlaying tracks of CUT&Tag peaks and RNA-Seq reads for SAG12, SAG16, and SAG18.

To understand the DNA-binding profile of EtAP2-S1, we conducted experiments using CUT&Tag and the C-terminal Flag-tagged EtAP2-S1 over-expressing strain. The enrichment experiments revealed that most EtAP2-S1 binding peaks were in close proximity to the transcription start site (TSS, Figure 6A). Furthermore, by overlapping two independent enrichments, a total of 2427 peaks associated with protein-coding genes were identified (Additional file 5). Additionally, 2246 peaks were located within a distance of 2 kb to the TSS and 2204 peaks were located within a distance of 1 kb to the TSS (Figure 6B, Additional file 5). A gene ontology enrichment analysis showed that these genes were significantly enriched for biological processes of gene expression (GO:0010467, q value = 0.015) and RNA metabolic process (GO:0016070, q value = 0.029) (Figure 6C).

Our results indicated that EtAP2-S1 binds to the upstream of 330 differentially expressed following its depletion, including 51 down-regulated and 279 up-regulated genes (Additional file 3). Notably, the promoters of 18 *sag* genes were bound by EtAP2-S1 (Additional file 3, Figures 6D–F), suggesting direct regulation of these genes. We also observed that two ApiAP2s (ETH2_1513500 and ETH2_0831900) were direct targets of EtAP2-S1. However, their roles require further investigation. The DNA-binding motif of EtAP2-S1 was identified using the DNA sequences under the peaks. Two motifs showed very high E-values, one being the repeat sequence “GCTGCT” (E-value = 2.1e−162) and the other “AAACCCT” (E-value = 2.4e−102). However, these motifs are strongly believed to be artificial due to the ubiquitous presence of “CAG” and telomere-like repeats in the genome of *Eimeria* species [43].

### EtAP2-S1-KO strain provides efficient immune protection against challenges

**Figure 7:**
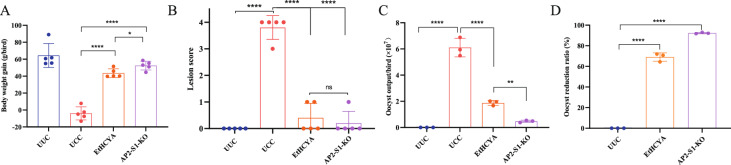
**EtAP2-S1 knockout parasite confers efficient immune protection.** Chickens were orally immunised with 1000 EtAP2-S1-KO or EtHCYA oocysts and were challenged with 2 × 10^4^ EtHCYA parent line at 14 dpi. The average body weight gain (**A**), cecal lesion scores (**B**), total oocyst output per bird (**C**), and oocyst reduction ratio (**D**) were measured and calculated. UUC, unimmunised and unchallenged control group; UCC, unimmunised and challenged group. **P < 0.01; ***P < 0.001; ****P < 0.0001; ns, non-significant.

Here, the immune protection efficiencies of the EtAP2-S1-KO and EtHCYA strains were compared to test the potential of the EtAP2-S1 knockout strain as a vaccine candidate. Chickens were immunised with 1000 fresh sporulated oocysts of each strain and challenged with a 2 × 10^4^ EtHCYA parent line. The average body weight gains (Figure 7A) and intestinal lesions (Figure 7B) in the immunised groups showed significant recovery compared to the challenge control. Additionally, the knockout strain showed a slightly higher average body weight gain than its parent strain immunisation (Figure 7A).

The total oocyst output after the challenge was also measured, demonstrating that immunisation significantly reduced the production of oocysts (Figure 7C). Surprisingly, EtAP2-S1-KO immunisation achieved a > 90% oocyst reduction rate, which was substantially higher than that of the EtHCYA immunised group (Figure 7D). Based on these parameters, the ACIs for the two strains were calculated to be 174.51 and 153.76 for the EtAP2-S1-KO and EtHCYA strains, respectively.

Our results, therefore, suggest that EtAP2-S1 knockout not only reduces parasite virulence but also partially enhances parasite immune protection efficiency. This finding makes EtAP2-S1 a promising vaccine candidate for chicken coccidiosis.

## Discussion

The *Eimeria* species is the causative agent of coccidiosis in animals, especially in poultry. Due to its huge economic burden on the poultry industry, coccidia is of significant veterinary importance. To a certain extent, the life cycle of coccidia is analogous to that of *Eimeria* and *Toxoplasma*, including schizogony and gametogony within a restricted definitive host. Therefore, the *Eimeria* model can be used as a substitute for studying the biology of *Toxoplasma* schizogony and sexual development, thereby eliminating the need to utilise cats. In this study, we characterised EtAP2-S1 and found that it binds to the promoters of multiple genes while directly suppressing *sag* gene expression. Furthermore, the depletion of EtAP2-S1 resulted in endogenous developmental deficiency and reduced virulence, while the gene-knockout strain showed strong potential as a vaccine candidate for coccidiosis.

Following the depletion of EtAP2-S1, over half of the up-regulated genes were highly expressed in the late merozoite (MZ120) and gametocyte stages (data not shown). Taking the up-regulated *sag* genes as an example, the majority of these genes were highly expressed in merozoites and gametes (Figure 5B). This result suggests that EtAP2-S1 suppresses the expression of merozoite and gamete genes, similar to the function of its ortholog, TgAP2XI-2, which inhibits the expression of numerous pre-sexual genes in *T. gondii* tachyzoites [22, 23]. The knockdown of TgAP2XI-2 and its partner TgAP2XII-1 results in a stage transition from the tachyzoite to the pre-sexual merozoite. Although the knockout of EtAP2-S1 significantly decreased parasite fitness, it did not affect the stage transition. EtAP2-S1-KO parasites still completed their entire life cycle without changing the prepatent time and with a similar pattern of oocyst output curve. These data suggest that EtAP2-S1 and TgAP2XI-2 play different roles in the coccidian life cycle and T. gondii tachyzoites, respectively.

However, similar to *Eimeria* SAGs, SAG-related sequence (SRS) genes are presumed to be *T. gondii* surface antigens containing a signal peptide and a predicted GPI anchor. Interestingly, EtAP2-S1 suppresses the expression of SAGs in the sporozoite stage, similar to how TgAP2IX-1 suppresses the expression of the *T. gondii* SRS gene in tachyzoites [44]. Notably, of greater interest is the role that TgAP2XI-2 plays in *T. gondii* in the cat enteroepithelial stages and whether it is similar to EtAP2-S1.

Moreover, EtAP2-S1 depletion leads to the up-regulation of 59 *sag* genes in sporozoites, most of which are merozoite-specific. Previous studies have shown that recombinant SAGs, including SAG1, SAG4, SAG7, SAG31, and SAG47, can bind to the surface of host cells in vitro [43]. In this study, SAG7 was up-regulated after EtAP2-S1 knockout, while the invasion efficiency and number of schizonts were significantly reduced in EtAP2-S1-KO parasites. It is crucial to investigate the precise functions of these SAGs further to provide a clearer explanation and understanding. Such an investigation will help clarify their roles in parasite attachment, invasion, and host-parasite interaction. On the other hand, however, the reduced invasive ability and fitness can be partly explained by the reduction of other fitness-conferring genes, such as the calmodulin-binding protein (ETH2_1323800) and Ca^2+^ ATPase (ETH2_0906100). *T. gondii* calmodulin-like proteins may interact with MyoH to control motility and cell invasion [45]. However, in a mouse model, Ca^2+^ ATPase-deficient *T. gondii* showed significantly decreased proliferation and virulence [46].

Within the current approach to coccidiosis control, attenuated live vaccines have proven effective and cost-efficient [47]. However, these vaccines are challenged by antigenic variation and strain-specific immunity, especially in *E. maxima* [48, 49]. With increased knowledge of virulent factors of *Eimeria* and the development of the CRISPR-Cas9 gene editing system, gene-knockout vaccines may provide more possibilities for coccidia control. For example, customised vaccines against different parasite-strain epidemics in different regions and countries. Consequently, this study generated a gene-knockout strain in *E. tenella* with reduced virulence while being capable of generating high protective immunity. These features make it a good vaccine candidate against coccidiosis.

In summary, we have demonstrated that EtAP2-S1 suppresses the expression of surface antigen genes, and its depletion leads to a significant decrease in parasite invasion and fitness. Moreover, the EtAP2-S1-KO strain exhibited low virulence and high immunogenicity, positioning it as a strong vaccine candidate. These results broaden our knowledge of gene regulation in coccidian parasites and provide the first step for developing a gene-knockout vaccine for controlling coccidiosis.

## Supplementary Information

**Additional file 1: Primers used in this study.**

**Additional file 2: Construction and identification of EtAP2-S1 overexpression (OE) parasite.** **A** Diagram showing the overexpression plasmid consisting of a DHFR-EYFP selection cassette and an overexpression cassette driven by the Actin promoter for the EtAP2-S1 copy. **B** Fluorescence microscopy image of EtAP2-S1-OE. **C** PCR identification of EtAP2-S1-OE. **D** Western blot identification of EtAP2-S1 overexpression. The total protein of the sporozoites of EtAP2-S1-OE and ETH strains were extracted for immunoblotting and detected by mouse anti-Flag antibody. Actin was used as a housekeeping control. **E** Oocyst output curves of EtAP2-S1-OE and ETH strains. Chickens (*n* = 5) were infected with 1000 fresh oocysts/bird for each strain, and oocyst outputs were monitored daily over a 5–12 dpi period. Total oocyst output was calculated for each bird (**F**). ns, non-significant.

**Additional file 3: RNA-Seq DEGs of EtAP2-S1 knockout strain compared to EtHCYA.**

**Additional file 4: Heatmaps of differentially expressed zinc map and gamete-related proteins after EtAP2-S1 knockout.**

**Additional file 5:** **The annotated CUT&Tag peak profile of EtAP2-S1.**

## Data Availability

Raw sequencing data and processed data for CUT&Tag and RNA-Seq experiments are available in the NCBI GEO database under the accession number GSE282226.
