# Supplementary figures and images for: Molecular mechanisms of surface antigen suppression by ApiAP2 and its implications for vaccine development
Source: Vet Res. 2025 Mar 22;56:63. doi: 10.1186/s13567-025-01491-2 (PMC11929185; doi:10.1186/s13567-025-01491-2)

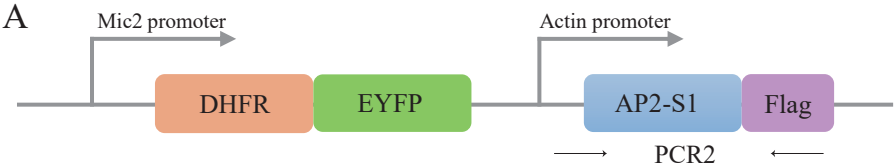

**B**

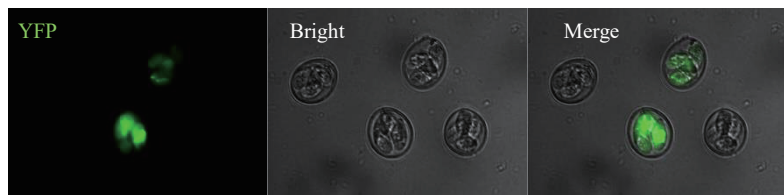

**C**

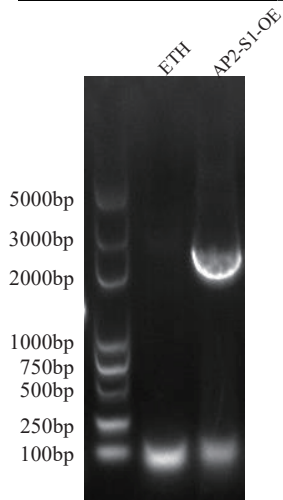

**D**

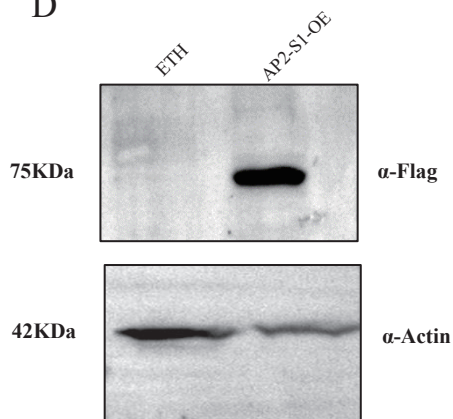

**E**

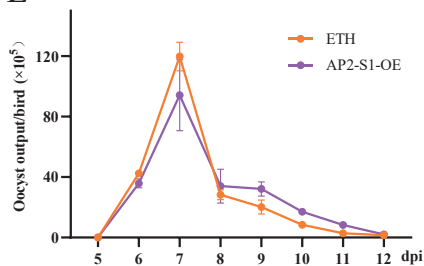

**F**

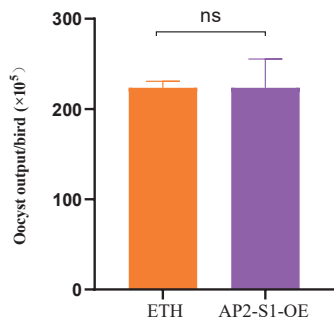

Supplement: Supplementary file 2 — Additional file 2: Construction and identification of EtAP2-S1 overexpression (OE) parasite. A Diagram showing the overexpression plasmid consisting of a DHFR-EYFP selection cassette and an overexpression cassette driven by the Actin promoter for the EtAP2-S1 copy. B Fluorescence microscopy image of EtAP2-S1-OE. C PCR identification of EtAP2-S1-OE. D Western blot identification of EtAP2-S1 overexpression. The total protein of the sporozoites of EtAP2-S1-OE and ETH strains were extracted for immunoblotting and detected by mouse anti-Flag antibody. Actin was used as a housekeeping control. E Oocyst output curves of EtAP2-S1-OE and ETH strains. Chickens (n = 5) were infected with 1000 fresh oocysts/bird for each strain, and oocyst outputs were monitored daily over a 5–12 dpi period. Total oocyst output was calculated for each bird (F). ns, non-significant. [file 13567_2025_1491_MOESM2_ESM.pdf]

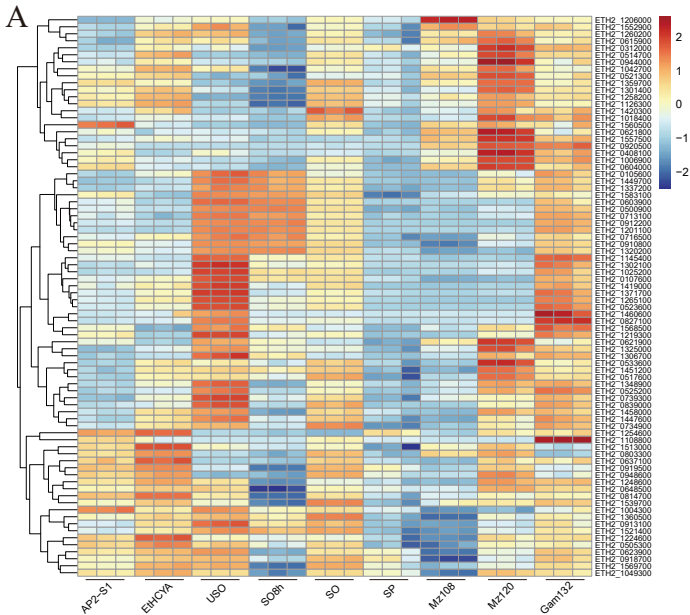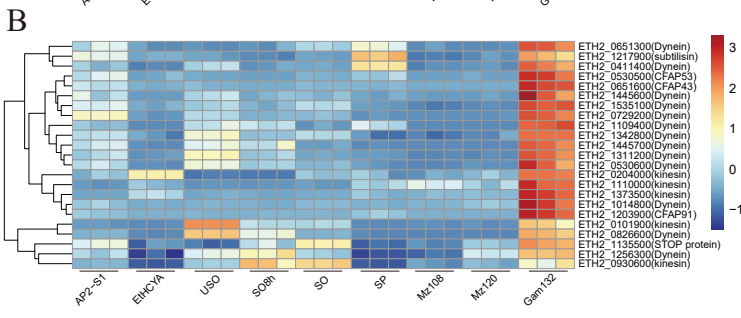

Supplement: Supplementary file 4 — Additional file 4: Heatmaps of differentially expressed zinc map and gamete-related proteins after EtAP2-S1 knockout. [file 13567_2025_1491_MOESM4_ESM.pdf]
